# Sexual Victimization in the Digital Age: A Population-Based Study of Physical and Image-Based Sexual Abuse Among Adolescents

**DOI:** 10.1007/s10508-021-02200-8

**Published:** 2022-01-20

**Authors:** Willy Pedersen, Anders Bakken, Kari Stefansen, Tilmann von Soest

**Affiliations:** 1 Department of Sociology and Human Geography, University of Oslo, Box 1096 Blindern, 0317 Oslo, Norway; 2 Section for Youth Research, Norwegian Social Research, OsloMet-Oslo Metropolitan University, Oslo, Norway; 3 Department of Psychology, University of Oslo, Oslo, Norway

**Keywords:** Sexual victimization, Sexual assault, Image-based sexual abuse, Adolescence, Social media

## Abstract

Adolescents increasingly use social media platforms, and these practices open up new forms of sexual victimization, in particular image-based sexual abuse (IBSA). Few studies have examined prevalence rates and correlates of both physical sexual victimization (PSV) and these new forms of victimization in representative samples. We used data from 5,245 adolescent girls (53%) and 4,580 adolescent boys (47%) from the population-based *Young in Oslo Study* (mean age 17.1 years, SD = 0.9). Of all respondents, 2.9% had experienced IBSA, 4.3% PSV, and 1.7% both IBSA and PSV in the course of the previous 12 months. Multivariate analyses revealed that PSV victims, after control for other variables, had many characteristics described in previous studies of sexual victimization. Girls had higher prevalence rates than boys, many had been victims of other types of violence, and were part of peer groups with much use of alcohol and drugs. PSV victims also reported early intercourse onset and a higher proportion had been commercially sexually exploited. Lesbian, gay, and bisexual adolescents had higher victimization rates. Victims of both PSV and IBSA had a similar but even more pronounced profile. The IBSA victims were different: They lacked many of the traditional risk factors for sexual victimization, there were no significant gender differences in this group, and IBSA victims more often came from high socioeconomic backgrounds. In conclusion, we observe a reconfigured landscape of sexual victimization patterns among Norway adolescents due to their increasing participation on social media and digital platforms.

## Introduction

Adolescents increasingly live “digital lives” (Suzor, 2019). However, research on sexual violence has been all too often “siloed”, with separate bodies of work on digital sexual victimization and physical sexual victimization. We will show how image-based sexual abuse (IBSA), now supplements physical sexual victimization (PSV), defined as sexual assaults by the use of force among adolescents. We study the prevalence and role of established risk factors of both phenomena. To this end, we use data from a large population-based sample of adolescents from Oslo, the capital of Norway.

### Image-Based Sexual Abuse

Adolescent sexuality increasingly includes activities in the digital arena. “Sexting” has become popular (Cooper et al., 2016; Crofts et al., 2016; Jonsson et al., 2015), and is defined as the consensual transmission of sexually written or photo/video material, usually via smartphones (Patrick et al., 2015). Whereas younger adolescents may jokingly exchange sexts with friends, much sexting activity among older adolescents seems to occur within committed relationships (Choi et al., 2019). The prevalence varies, owing to differences in definitions, criteria, and samples used (Döring, 2014; Drouin & Tobin, 2014). However, a recent meta-analysis (Madigan et al., 2018), identified 39 relevant studies (participants under 18 years, mean age 15.2 years, a majority from the USA and Europe). An average of 15% had sent and 27% had received sexts. The prevalence rate has been rising, reflecting the increasing use of smartphones.

Researchers have suggested that sexting practice, even if seemingly consensual, may lead to non-voluntarily sexting in response to pressure as well as lack of consent for the forwarding of images (Wastler, 2010). For clearly non-consensual sharing of sexual images, new definitions are emerging, and IBSA now seems to be the most common term (Harder et al., 2019). IBSA behaviors are often divided into two main groups (Drouin & Tobin, 2014). The first is “sexting coercion,” often defined as pressuring someone to take part in unwanted interaction via sexually explicit texts, pictures, or videos. The second implies the distribution of sexually explicit pictures or videos of another person to third parties without this person’s consent. We concentrate on the latter behavior.

IBSA contexts include relationship retribution (misuse of images of a partner), sextortion (threats to distribute images to obtain benefits), and sexploitation (with the goal of obtaining monetary benefits) (Powell et al., 2018). An online study from Australia (age range 16–49 years) is so far the most comprehensive study of IBSA perpetrators (Powell et al., 2019). One in ten self-reported some form of IBSA perpetration, more males (14%) than females (7%). The lesbian, gay, or bisexual (LGB) group had almost three times as high prevalence of perpetrators (20%) as those who identified themselves as heterosexual (7%). Among victims of IBSA, there were also a substantially higher prevalence of perpetrators as opposed to those without IBSA victim experiences. The victim was typically an intimate or ex-partner, family member, or friend. However, the response rate in this study was only 3.8%, and one may question whether the prevalence (10%) was inflated due to a selected sample. Other studies suggest that just under 10% have experienced any form of IBSA, and a lesser number (3–5%) report nonconsensual distribution (Eaton et al., 2018; Harder et al., 2019; Powell & Henry, 2019).

Nonconsensual distribution of sexual images has been framed as a form of gender-based sexual abuse for which women are usually targets (Henry & Powell, 2015). However, a review suggests the findings are mixed when it comes to gender and IBSA—with some studies suggesting that in adult samples, males are more often victims—whereas the opposite may be the case among adolescents (Walker & Sleath, 2017). Nevertheless, such experiences may be gendered in specific ways, and in a recent study, females were more often exposed to sexual harassment and explicit images (e.g., “dick pics”), whereas males were more often victims of nonconsensual distribution of sexual images (Powell & Henry, 2019).

### Physical Sexual Victimization

PSV among adolescents has been researched for decades. Here, the gender differences are more consistent than those observed in the digital landscape, with girls being more commonly victimized than boys (for a review, see Barth et al., 2013). Furthermore, PSV seems to be more prevalent in adolescent age groups than among adults (Young et al., 2009). The majority of adolescent sexual assaults seems to be committed by an acquaintance of the victim (Rennison, 2002), often in the context of dating or a stable relationship (Taylor & Mumford, 2016), even though other areas such as school (Tillyer et al., 2010) and workplace may also be important (Sears et al., 2011).

A population-based study of adolescents from the USA revealed that 8% reported a history of forced sexual intercourse, twice as many girls as boys (Howard et al., 2007). In a study using a high school sample, this was true for 12% of girls and 3% of boys (Young et al., 2009). In a recent study of Norwegian high school students aged 18–19 years (Stefansen et al., 2020b), utilizing a variety of criteria of serious and forced sexual victimization and rape, 14% of the girls had experienced physical sexual victimization, as opposed to 3% of the boys.

### Risk Factors for Adolescent Sexual Victimization

While few studies have identified risk factors for IBSA, numerous studies have investigated risk factors for PSV. The latter has increasingly been conceptualized in broader developmental models (Matta Oshima et al., 2014), including family risk factors such as socioeconomic status, parental education, parental divorce, and poor parental care and monitoring (Assink et al., 2019). Therefore, in the current study, we identify correlates of IBSA and PSV in several domains. However, as adolescent sexual victimization is often peer-related (Rennison, 2002), we focus in particular on correlates related to the peer milieu of adolescents (Lorenz & Ullman, 2016; Pittenger et al., 2016; Taylor & Mumford, 2016).

#### Bullying and Sexual Violence Exposure

Several studies suggest that milieus with interpersonal violence are associated with sexual victimization (Ports et al., 2016). However, in much of the literature on violence and bullying, sexual victimization is peripheral or absent (Owens et al., 2005). Nonetheless, some studies have suggested that there are complex border zones between violence, bullying, and sexual harassment and that researchers may have overlooked the importance of sexualized elements in boys’ bullying of girls (Shute et al., 2008). Thus, we explore whether a social environment with much bullying and violence may also place adolescents at higher risk of IBSA and PSV.

#### Cyber Activities and Aggression

Cyber aggression seems to co-occur with sexual partner violence in adult samples (Marganski & Melander, 2018). We know less about the impact of ordinary cyber activities among adolescents on either IBSA or PSV. For example, excessive social media use may have negative consequences; adolescents may be encouraged to engage in risky behaviors frequently displayed in social media, such as binge drinking and high-risk sexual behaviors (Chassiakos et al., 2016). Thus, we explore whether exposure to cyber aggression and bullying and more generally spending excessive time on social media and developing relationships restricted to digital contexts, are correlates of IBSA or PSV.

#### Alcohol Use, Substance Use, and Partying

Often, physical sexual assaults among young people involve victims, perpetrators, or both who consume alcohol, typically in large quantities, before the assault (Lorenz & Ullman, 2016). Alcohol consumption may increase the likelihood of sexual assault through multiple pathways, including alcohol’s psychological, cognitive, and motor effects (Abbey, 2002, 2011). Consistent with routine activity theory, heavy drinking related to social contexts, such as parties, entails the convergence of risky situations, potential victims, and lack of guardians (Mustaine & Tewksbury, 2002). Combining alcohol with other psychoactive substances, such as cannabis, may increase the risk additionally (Mallett et al., 2017). A Norwegian study reported that 7% of adolescent girls who had been intoxicated with alcohol in the previous year had experienced sexual assault when incapacitated by drunkenness (Pape, 2014). Another Norwegian study, using the same measure, found a similar rate for slightly older girls, and a rate of 2% among boys (Stefansen et al., 2020a). Thus, we investigate potential links between both types of victimization and the use of alcohol and illegal substances.

#### Sexual Activities and Sexual Identity

A final group of potential correlates relates to more proximal sexuality-related risk factors. Early intercourse debut has been linked to having sex under the influence of alcohol and drugs, lack of use of contraceptives (Sandfort et al., 2008) as well as increased risk of sexual victimization (Kaplan et al., 2013). One explanation may be that sexual experiences “out of phase” with other peers often occur in contexts with little control (Impett & Peplau, 2003). Moreover, adolescents who are commercially sexually exploited (who obtain benefits for sexual favors) are particularly vulnerable (Pedersen & Hegna, 2003). One final antecedent of sexual victimization is having or exploring a non-heterosexual identity, (e.g. identifying as LGB). LGB youth experience higher levels of all types of sexual victimization (Balsam et al., 2005; Mellins et al., 2017; Toomey & Russell, 2016). This may be related to lack of support in the sexual identity exploration phase, as well as discrimination, invisibility, and rejection by family members (see Balsam et al., 2005). Above, we have also presented research findings suggesting that the LGB group has a higher proportion of digital perpetrators (Powell et al., 2019).

We explore the importance of all four groups of potential correlates for sexual victimization. To our knowledge, no previous studies have compared the correlates of IBSA and PSV.

### The Context

In Norway, 98–99% of 12–13 years old have their own smartphone. Around 80% of adolescents are active on social media (Norwegian Media Authority, 2019). Whereas 55% of the global population has Internet access, this is true for 99% of the Norwegian population (Statista, 2020). Thus, Norway is well suited for exploring sexual victimization related to digital media.

Norway also represents an interesting policy context for the study of sexual victimization among adolescents. Over the recent decades, there has been a marked increase in public expenditure on services and prevention programs related to sexual violence. This policy has been based on international conventions from the UN and the Council of Europe that Norway has implemented and that highlight the state’s responsibility to prevent all forms of gender-based violence (Skilbrei et al., 2020). It also reflects how sexual violence to an increasing degree is considered to harm not only the individual but also society at large. However, one should note that digital and physical sexual violence so far has been separated at the policy level in Norway and that most policy development still relates to the latter phenomenon. The same is the case for other countries in the Nordic region (Frøyland et al., 2021). However, this situation seems to be changing in Norway, as state authorities now are commissioning several studies on digital sexual violence and working on a national strategy against sexual exploitation of children and youths through digital media to be released in 2021.

### Research Questions and Hypotheses

First, we ask: what proportion of the adolescent population report IBSA, PSV, or both types of victimization? Are there differences by gender? Are there sociodemographic differences? Second: what correlates are revealed between these types of victimization and (a) traditional bullying and violence, (b) a variety of cyber activities, (c) substance use and partying, and (d) sexual behaviors and identifying as LGB?

## Method

### Participants

We use data from the *Young in Oslo Study* conducted in 2018. This is a school-based survey of living conditions, well-being, and a variety of risky behaviors among students in grades 8–13. We use data from students in upper secondary school in grades 11–13, covering birth cohorts born 1999 to 2001 (Bakken, 2018). In this age group, 95% of the total population attend high school. All of the city’s 24 public schools and four of nine private schools participated in the study. An electronic questionnaire was administered under the supervision of teachers in the classroom. The response rate was 65% and comprised around 60% of the total population of the included cohorts. The mean age was 17.1 years (SD = 0.9). While 5% had turned 19 years old, 16 to 18-year-olds were almost equally distributed. 53% of the respondents were girls and 33% of the respondents had immigrant background, compared to 38% in the population. All students were informed about the purpose of the study and told that participation was voluntary and that they could skip questions they did not want to answer. The study was approved by the Norwegian Data Protection Authority.

The analyses are based on a sample of 9,858 students who answered the questions about sexual victimization (i.e., IBSA and PSV). Of all respondents, 620 (5,9%) did not answer these two questions, most of whom stopped answering before these questions were introduced in the questionnaire. Boys and ethnic minority students were overrepresented among non-responders.

### Measures

#### Physical Sexual Victimization and Image-Based Sexual Abuse

Based on a review of instruments measuring forcible PSV (Davis et al., 2014), we asked whether the participant had “been forced to participate in intercourse or other sexual acts.” Regarding IBSA, we wanted to include images produced by the victim or the perpetrator and typically distributed after a break-up, as well as images produced by other persons without consent (e.g., videos of sexual encounters at parties taken by a third person, and “upskirting”: images of someone’s pubic area taken underneath their outer clothing; McGlynn & Rackley, 2017). Thus, we asked whether the respondent had experiences of someone who “against your will, has distributed pictures or videos in which you are naked or taking part in sexual acts.” For both questions, we asked about such experiences in the course of the past 12 months. The response options were *never*, *once*, *2–5 times*, and *6 times or more*.

#### Family Background

Socioeconomic background was measured by a composite socioeconomic score based on the average scores on three variables, which were all coded on a scale ranging from 0 to 3 (Pedersen, Bakken, & von Soest, 2017). We use data on (1) the number of parents who had a university degree, (2) the number of books in the home of the respondent (on a six-point scale from 0 to 1000+) (Sullivan, 2001), and (3) the average score on the four-item Family Affluence Scale-II (Currie et al., 2008). The Family Affluence Scale includes items assessing the number of computers and cars in the family, how many times the family had gone on holiday in the previous year, and whether the respondents had their own room at home. Parental participation in the labor market was measured by asking whether the parents are working. A marginal position was defined as not having at least one parent in full-time work. Immigrant background was measured by asking where parents were born and defined as having two parents born abroad. Family structure was measured by asking: “Which adults do you live with?” Respondents answering “both parents” were coded 0, others were coded 1.

Following Olweus (1989), we measured parental monitoring by asking how well three statements about whether their parents usually know where they are during leisure time, with whom they spend time, and whether their parents know their friends’ parents fit. Response options for all three items ranged from 0 (*fits very badly*) to 3 (*fits very well*), and mean scores were constructed where higher scores indicated a higher degree of parental monitoring. A measure of parental support was developed based on Sarason’s Social Support Scale (Sarason, Levine, Basham, & Sarason, 1983), where we used the question: “Imagine you have a personal problem. You feel out of place and sad and need someone to talk to. Would you talk to or seek help from your parents?” The response option *for sure* was coded 1, while *maybe* and *no* were coded 0.

#### Bullying and Violence

We measured bullying by asking “Have you been harassed, threatened, or ostracized by other young people at school or in your spare time?” (see Bjereld et al., 2020). Those who had experienced such harassment every fortnight or more frequently were coded 1, while others were coded 0. Exposure to violence was measured by asking how many times the respondents had experienced “Punches or kicks that did not lead to visible marks,” “Violence that led to bruises or injuries without the need for medical care,” or “Being so badly injured by violence that you needed medical care” during the previous year (Hamby & Turner, 2013). Response alternatives included *never* (0), *once* (1), *2–5 times* (2), and *6 times or more* (3). Mean scores across the three items were constructed. Family violence was measured using the question: “During the past year, have any of the adults in your family hit you on purpose?” (Jirapramukpitak et al., 2011). Response options ranged from *Never* (0) to *10 times or more* (4).

#### Cyber Activities

Daily time spent on social media was measured by asking “Imagine an average day. How much time do you spend on social media (Facebook, Instagram, etc.)” with response options ranging from *No time* (0) to *More than 3 h* (5). Cyber friendship was measured by a question about whether the respondents had at least one close friend with whom they only connected online. Affirmative answers were coded 1, while others were coded 0. Exposure to cyberbullying was modeled on previous research (Patchin & Hinduja, 2010) using three items about whether in the last few months, someone online or through their personal mobile phone had (1) threatened them, (2) excluded them from online social activities, or (3) written or published hurtful things about them online. Response options were *Never* (0), *Once* (1), *2–5 times* (2), and *6 times or more* (3). An average score across the three items was constructed.

#### Substance Use and Exposure to Substances

Heavy episodic drinking was measured by the question: “During the past year, how often have you drunk so much that you felt clearly intoxicated?” (Pedersen & von Soest, 2013). Response options ranged from *Never* (0) to *More than 10 times* (4). We asked about *cannabis use* (the most prevalent illegal drug in Norway) in the same way with the same response options. Use of other drugs was measured by a similar question about the use of MDMA, amphetamine and cocaine, the most prevalent other illegal drugs used among young people in Norway (Norwegian Institute of Public Health, 2018). Two items about whether the respondent had socialized with other young people who had drunk alcohol or used cannabis during the last 4 weeks measured exposure to peers’ substance use. Response options ranged from *Never* (0) to *five times or more* (3), and mean scores were computed.

#### Sexual Behaviors and Sexual Identity

Early sexual intercourse was measured by asking about age of first sexual intercourse. Not having had sexual intercourse or a debut age of 16 years or more (the legal age for consent in Norway) was coded 0, while intercourse debut at the age of 15 years or younger was coded 1. Commercial sexual exploitation is often measured by asking whether adolescents have “been paid by someone to have a sexual relationship with them” (Reid & Piquero, 2014). However, this question may be too narrow to capture all forms of such exploitation. Thus, we asked: “During the past 12 months, have you engaged in sexual acts in order to receive something in return (money, clothes, make-up, alcohol or drugs, accommodation, transport, food, or other things or gifts)?” with the response options *No* (0) and *Yes* (1). Drawing on previous research (Toomey & Russell, 2016), we measured sexual identity by asking whether the respondents considered themselves to be heterosexual or gay/lesbian. The responses *Gay/lesbian*, *A bit of both*, and *Not sure what label describes me* were coded 1, while all other answers were coded 0.

### Statistical Analysis

Sexual victimization experiences over the past 12 months were divided into four categories: (1) no victimization experiences, (2) IBSA only, (3) PSV only, and (4) both IBSA and PSV. We first tested gender differences in prevalence using chi-square and ANOVA tests by comparing each category with the rest of the sample. A series of bivariate multinomial logistic regression analyses were then used to investigate whether respondents in the three victimization categories differed from those who had no victimization experiences on all study variables. Finally, we included all independent variables simultaneously in one multinomial regression analysis to examine the unique contribution of each variable on group membership. We also tested possible interaction effects between gender and all independent variables. To handle missing data, we used a multiple imputation model with 10 imputations including all predictor variables. Because the results did not differ substantially from using listwise deletion, we report listwise deletion results. In order to reduce risk related to multiple testing and risk of Type 1 errors, we set the level of significance at *p* < 0.01. Data were analyzed using IBM SPSS 27.

## Results

The results showed that 4.6% of the sample had experienced IBSA, while 6.0% had experienced PSV over the past 12 months. Of all girls, 4.8% reported IBSA, compared to 4.3% of all boys (*p* = 0.13). Moreover, 8.3% of girls reported PSV, compared to 3.3% of boys (*p* < 0.001). Furthermore, Table 1 reveals that 2.9% had experienced IBSA only, 4.3% PSV only, and 1.7% both types of victimization. There were no significant gender differences for IBSA only, whereas girls were victims of PSV only almost three times as often as boys (*p* < 0.001). Girls were also overrepresented among those who had experienced both IBSA and PSV (*p* < 0.01).

**Table 1:** Prevalence of image-based sexual abuse (IBSA) and physical sexual victimization (PSV) during the previous 12 months among 16–19 year old youth in Oslo, Norway by gender

	Boys *(n* = *4580)*		Girls *(n* = *5245)*		Total *(N* = *9825)*		Test of gender difference	
	*n*	%	*n*	%	*n*	%	χ^2^	*p*
No experience of sexual victimization	4,291	93.7	4,662	88.9	8,953	91.1	69.8	< .001
IBSA only	138	3.0	147	2.8	285	2.9	0.4	.55
PSV only	92	2.0	330	6.3	422	4.3	109.1	< .001
Both IBSA and PSV	59	1.3	106	2.0	165	1.7	8.0	< .01

In Table 2, we present descriptive statistics for all independent variables for all victimization categories. The table shows a general pattern of higher frequency of being bullied and violence experiences, substance use, risky sexual activities, and LGB orientation among those who reported some form of sexual victimization (IBSA, PSV, or both) compared to those who did not report such experiences.

**Table 2:** Descriptive statistics of all study variables according to sexual victimization experiences

	No sexual victimization experiences (*n* = 8,953)		IBSA (*n* = 285)		PSV (*n* = 422)		Both IBSA and PSV (*n* = 165)		Total (*n* = 9,825)		*p*
	Mean	SD	Mean	SD	Mean	SD	Mean	SD	Mean	SD	
*Sociodemographics and family background*											
Female gender (%)	52.1%		51.6%		78.2%		64.2%		53.4%		< .001
Age (16–19 years)	17.08	0.89	17.05	0.88	17.19	0.91	17.03	0.86	17.09	0.89	.08
Parental socioeconomic status (0–3)	2.07	0.66	2.32	0.52	2.08	0.62	1.83	0.72	2.07	0.65	< .001
Marginalized parental position in labor market (%)	9.2%		3.5%		9.9%		14.8%		9.2%		< .001
Immigrant background	31.0%		12.0%		22.0%		38.0%		30.0%		< .001
Not living with both parents (%)	34.1%		38.4%		49.2%		46.0%		35.1%		< .001
Parental monitoring (0–3)	2.38	0.64	2.24	0.67	2.17	0.72	2.02	0.77	2.36	0.65	< .001
Close relation to parents (%)	40.3%		31.6%		26.7%		28.1%		39.2%		< .001
*Bullying and violence*											
Regularly bullied (%)	3.5%		7.0%		12.4%		27.5%		4.4%		< .001
Exposed to physical violence (0–3)	0.10	0.32	0.26	0.53	0.32	0.55	0.74	0.85	0.12	0.36	< .001
Family violence (%)	6.5%		13.0%		17.6%		30.1%		7.5%		< .001
*Digital lives*											
Time spent on social media (0–5)	3.26	1.40	3.67	1.21	3.90	1.18	3.78	1.44	3.31	1.39	< .001
Has at least one cyberfriend (%)	36.8%		35.4%		40.5%		53.7%		37.2%		< .001
Exposed to cyberbullying (0–3)	0.22	0.45	0.52	0.67	0.64	0.72	1.04	0.86	0.26	0.50	< .001
*Substance use*											
Alcohol intoxication (0–4)	1.39	1.54	2.66	1.38	2.39	1.47	2.28	1.57	1.48	1.56	< .001
Cannabis use (0–4)	0.55	1.14	1.24	1.46	1.14	1.43	1.19	1.50	0.60	1.18	< .001
Other narcotics use (0–4)	0.04	0.26	0.09	0.39	0.14	0.48	0.48	1.06	0.06	0.32	< .001
Peers’ substance use (0–3)	1.09	0.99	1.82	0.88	1.76	0.92	1.69	1.09	1.15	1.00	< .001
*Risky sexual activities and sexual identity*											
Early sexual intercourse (% < 16 years old)	29.8%		58.4%		66.2%		65.6%		32.8%		< .001
Benefits in exchange for sex (%)	2.8%		7.9%		14.5%		31.5%		3.9%		< .001
Sexual orientation (% LGB)	9.0%		11.2%		21.3%		20.9%		9.8%		< .001

*IBSA* = image-based sexual abuse; *PSV* = physical sexual victimization; *LGB* = lesbian, gay, or bisexual

In Table 3, we present odds ratios (*OR*) from bivariate multinomial regressions. Experiences of PSV and both PSV and IBSA were substantially more prevalent among girls than boys, whereas no significant gender differences were found for IBSA only. Those who had experienced IBSA only were more often from families with higher socioeconomic status compared to the rest of the sample. Overall, for most potential risk variables, all three victimization groups showed higher scores. Despite such similarities, the PSV group was more exposed to typical correlates of sexual victimization such as bullying experiences, risky sexual activities, and LGB orientation, compared to the IBSA-only group. However, the group that had experienced both IBSA and PSV had the highest ORs in the domains of bullying and violence, illicit drug use, and commercial sexual exploitation.

**Table 3:** Bivariate logistic regression models with sexual victimization types as the dependent variable, and no sexual victimization experiences as the reference category

	IBSA only versus no sexual victimization experiences				PSV only versus no sexual victimization experiences				Both IBSA and PSV versus no sexual victimization experiences			
	OR	95% CI		*p*	OR	95% CI		*p*	OR	95% CI		*p*
*Sociodemographics and family background*												
Gender (female = 1)	0.98	0.77	1.24	.87	3.30	2.61	4.18	< .001	1.65	1.20	2.28	< .01
Age	0.95	0.83	1.09	.46	1.14	1.02	1.27	.02	0.93	0.78	1.11	.43
Socioeconomic status (0–3)	2.04	1.64	2.54	< .001	1.02	0.88	1.18	.82	0.60	0.48	0.74	< .001
Parents’ position in labor market (marginal = 1)	0.36	0.18	0.69	< .01	1.09	0.77	1.52	.64	1.71	1.06	2.77	.03
Immigrant background (two immigrant parents = 1)	0.30	0.21	0.43	< .001	0.63	0.50	0.80	< .001	1.35	0.98	1.86	.07
Family structure (not living with both parents = 1)	1.20	0.93	1.55	.16	1.87	1.53	2.29	< .001	1.64	1.17	2.31	< .01
Parental monitoring (0–3)	0.73	0.62	0.87	< .001	0.65	0.57	0.74	< .001	0.50	0.41	0.61	< .001
Close relation to parents (1 = yes)	0.69	0.53	0.89	< .01	0.54	0.43	0.67	< .001	0.58	0.41	0.83	< .01
*Bullying and violence*												
Regularly bullied (1 = yes)	2.06	1.29	3.29	< .01	3.86	2.83	5.27	< .001	10.35	7.19	14.91	< .001
Exposed to physical violence (0–3)	2.52	2.02	3.16	< .001	2.95	2.47	3.52	< .001	6.01	4.91	7.34	< .001
Family violence (1 = yes)	2.14	1.47	3.12	< .001	3.05	2.32	4.02	< .001	6.17	4.22	9.03	< .001
*Digital lives*												
Time spent on social media (0–5)	1.25	1.14	1.37	< .001	1.45	1.34	1.57	< .001	1.34	1.18	1.53	< .001
Cyberfriend (yes = 1)	0.94	0.74	1.20	.63	1.17	0.96	1.43	.13	1.99	1.46	2.71	< .001
Exposed to cyberbullying (0–3)	2.52	2.12	2.98	< .001	3.10	2.71	3.54	< .001	5.12	4.29	6.11	< .001
*Substance use*												
Alcohol intoxication (0–4)	1.67	1.54	1.81	< .001	1.49	1.40	1.59	< .001	1.42	1.29	1.57	< .001
Cannabis use (0–4)	1.45	1.34	1.56	< .001	1.39	1.30	1.48	< .001	1.42	1.28	1.57	< .001
Other narcotics use (0–4)	1.55	1.17	2.07	< .01	1.90	1.57	2.31	< .001	2.98	2.50	3.56	< .001
Peers’ substance use (0–3)	2.04	1.81	2.31	< .001	1.92	1.74	2.12	< .001	1.79	1.54	2.10	< .001
*Risky sexual activities and sexual identity*												
Early sexual intercourse (< 16 years old = 1)	3.30	2.59	4.21	< .001	4.61	3.75	5.68	< .001	4.50	3.24	6.25	< .001
Benefits in exchange for sex (yes = 1)	2.97	1.85	4.78	< .001	5.88	4.30	8.04	< .001	16.01	10.81	23.71	< .001
Sexual orientation (LGB = 1)	1.27	0.86	1.88	.23	2.73	2.12	3.52	< .001	2.66	1.74	4.07	< .001

*IBSA* = image-based sexual abuse; *PSV* = physical sexual victimization; *OR* = odds ratio; *CI* = confidence interval; *LGB* = lesbian, gay, or bisexual

In Table 4, we present the results of multinomial regression models, where all independent variables were included simultaneously in one analysis. When all variables were controlled for, girls still had significantly higher rates of PSV only and of both PSV and IBSA, while gender was not associated with IBSA only. Generally, background and family factors were not associated with any victimization category. An exception was the remaining significant association between high socioeconomic status and experiencing IBSA only. IBSA only was also associated with excessive use of social media, cyberbullying, and alcohol intoxication. No other variables were associated with IBSA only.

**Table 4:** Multinomial logistic regression models with sexual victimization types as the dependent variable, and no sexual victimization experiences as the reference category

	IBSA only versus no sexual victimization experiences				PSV only versus no sexual victimization experiences				Both IBSA and PSV versus no sexual victimization experiences			
	OR	95% CI		*p*	OR	95% CI		*p*	OR	95% CI		*p*
*Sociodemographics and family background*												
Gender (female = 1)	1.13	0.83	1.53	.44	5.11	3.74	6.98	< .001	4.21	2.48	7.15	< .001
Age	0.89	0.76	1.04	.13	1.05	0.92	1.20	.45	0.66	0.51	0.85	< .001
Socioeconomic status (0–3)	1.61	1.18	2.18	< .01	0.94	0.75	1.18	.58	0.76	0.52	1.12	.16
Parents’ position in labor market (marginal = 1)	0.50	0.22	1.15	.10	1.27	0.83	1.93	.27	1.57	0.79	3.14	.20
Immigrant background (two immigrant parents = 1)	0.66	0.41	1.07	.09	0.88	0.63	1.24	.47	1.23	0.71	2.16	.46
Family structure (not living with both parents = 1)	1.00	0.75	1.33	.98	1.09	0.86	1.40	.48	0.84	0.53	1.33	.46
Parental monitoring (0–3)	0.87	0.70	1.08	.19	0.90	0.75	1.07	.22	0.90	0.66	1.22	.49
Close relation to parents (1 = yes)	0.95	0.71	1.28	.74	0.79	0.61	1.03	.09	1.14	0.69	1.88	.61
*Bullying and violence*												
Regularly bullied (1 = yes)	1.18	0.66	2.11	.58	1.70	1.11	2.60	.02	1.82	0.97	3.43	.06
Exposed to physical violence (0–3)	1.32	0.96	1.80	.09	1.55	1.18	2.02	< .001	2.36	1.66	3.37	< .001
Family violence (1 = yes)	1.67	1.08	2.57	.02	1.46	1.02	2.10	.04	1.78	1.03	3.07	.04
*Digital lives*												
Time spent on social media (0–5)	1.16	1.04	1.31	< .01	1.13	1.02	1.25	.02	1.10	0.92	1.31	.31
Cyberfriend (yes = 1)	1.07	0.79	1.44	.67	1.21	0.94	1.55	.14	1.30	0.83	2.04	.25
Exposed to cyberbullying (0–3)	1.87	1.49	2.35	< .001	2.00	1.65	2.42	< .001	2.80	2.08	3.77	< .001
*Substance use*												
Alcohol intoxication (0–4)	1.29	1.15	1.46	< .001	1.18	1.07	1.31	< .001	1.44	1.19	1.73	< .001
Cannabis use (0–4)	1.07	0.96	1.21	.23	1.05	0.95	1.17	.34	0.87	0.72	1.06	.18
Other narcotics use (0–4)	0.75	0.46	1.25	.27	1.21	0.88	1.68	.24	2.06	1.40	3.04	< .001
Peers’ substance use (0–3)	1.24	1.04	1.49	.02	1.34	1.15	1.56	< .001	1.12	0.86	1.47	.40
*Risky sexual activities and sexual identity*												
Early sexual intercourse (< 16 years old = 1)	1.48	1.10	1.99	< .01	2.42	1.86	3.16	< .001	3.30	1.96	5.55	< .001
Benefits in exchange for sex (yes = 1)	1.53	0.90	2.61	.12	2.19	1.47	3.26	< .001	3.84	2.21	6.68	< .001
Sexual orientation (LGB = 1)	1.26	0.82	1.92	.30	1.92	1.42	2.59	< .001	2.03	1.18	3.49	< .01

*IBSA* = image-based sexual abuse; *PSV* = physical sexual victimization; *OR* = odds ratio; *CI* = confidence interval; *LGB* = lesbian, gay, or bisexual

When comparing PSV only victims with those who had not experienced any victimization, we observed more significant associations, such as exposure to bullying and violence, being in social settings where other young people used substances, and engaging in risky sexual behaviors (early intercourse, commercial sexual exploitation). In addition, LGB youth had higher prevalence of PSV. The multivariate analysis confirmed the findings from the bivariate analysis regarding those who had experienced both IBSA and PSV: This group was similar to the PSV only group, showing higher prevalence of physical violence, alcohol intoxication, use of illegal substances, as well as early intercourse debut, commercial sexual exploitation, and LGB identification, compared to those with no victimization experiences. Of note, also in multivariate analyses, *OR* were in most cases higher for the IBSA and PSV group, compared to the PSV only group.

In a final set of analyses, we examined whether associations between all predictor variables and victimization groups were moderated by gender by including interaction terms in multinomial logistic regression analyses. Of the 60 interaction terms tested in these analyses, two were significant at *p* < 0.01. The results thus provide no clear indications of gender-specific effects over and above what would be expected by chance because of multiple testing.

## Discussion

Drawing on a population-based sample of Norwegian adolescents, we have described a reconfigured landscape of sexual victimization. Almost as many are now victims of image-based sexual abuse (IBSA) as of physical sexual victimization (PSV). Thus, the total number of adolescents experiencing sexual victimization may have increased with the influx of digital forms of sexual victimization.

The PSV group in our study showed many characteristics mirroring previous research of sexual victimization, and our study shows similar results for adolescents experiencing both PSV and IBSA. In the latter group, several of these characteristics were—both before and after statistical control—even more pronounced than in the PSV only group. In both these groups, girls were more often victims than boys. Experiences of physical violence, illegal drug use, and peer networks involving substance use were more prevalent, the same was early intercourse debut and commercial sexual exploitation. Moreover, LGB youth had higher rates of such victimization.

The IBSA only group had a somewhat different profile: First, as many boys as girls reported IBSA only experiences. Second, IBSA victims had on average higher SES backgrounds than those without victimization experiences. Third, they were not characterized by commercial sexual exploitation and LGB youth did not have increased rates of IBSA.

We suggest that the findings contribute to new insights regarding what Khan et al. (2020) call “the social organization of sexual assault”. The driving force is the rapid development of adolescents’ digital surroundings and interactions. Almost all Norwegian adolescents are active on social media. Facebook used to be the most important platform, then Snapchat, YouTube, and Instagram were introduced, while TikTok has gained popularity over the past couple of years (Norwegian Media Authority, 2019). These platforms open up different and unique types of sexual assaults (Anderson, 2020). Related to this, the widespread practice among youth of documenting and sharing intimate aspects of life through social media can lower the bar for digital sexual transgressions as such practices may be interpreted as part of the “normal” or as something insignificant.

The gender-equal distribution of IBSA that has been described in handful of other studies (Walker & Sleath, 2017) is a key finding in our study. This means that the gendering of sexual victimization may be formulated differently in the digital landscape than an in-person physical victimization. However, even within comparably gender-equal “hook-up cultures” such as in Norway, heterosexual boys may be expected to be sexually active, while a double standard is evident in that sexually active girl’s easier risk stigmatization through “bad reputation” (Fjær et al., 2015). Against this background, we hypothesize that the meaning of IBSA may differ between the two genders and that IBSA—for girls—may more often be contextualized as part of a traditional pattern of “slut-shaming.” For boys, the distribution of explicit sexual material from a party context may be interpreted as contributing to a reputation as “a player” (Boogle, 2008, pp. 104–105), even if there may be fluid boundaries to the more stigmatized “fuckboy” category. Girls seem to be held responsible more often than boys if images are shared, as they may have—more or less—voluntarily sent images to a boyfriend in the first place (Dobson & Ringrose, 2016), partly because they also feel more pressure than boys to engage in such behavior (Drouin & Tobin, 2014). Thus, it will be important to investigate whether IBSA may have consequences that are more serious for girls than for boys. In line with this, a recent study in Norway suggested that girls found sexualized online comments to be more threatening than boys (Norwegian Media Authority, 2019). Thus, while boys and girls are equally at risk of IBSA, there may be gendered dimensions to the dynamics and effects of such experiences.

One should note that an LGB identity was not associated with higher rates of IBSA in our study; however, these adolescents had clearly increased rates of PSV and both PSV and IBSA. Previous research suggests that this may be because LGB youth experience prejudice, discrimination, invisibility, and rejection by family members (see Balsam et al., 2005).

Another difference between IBSA and PSV relates to socioeconomic background. In contrast to victims of PSV (Phipps, 2009), IBSA victims tended in our study to come from higher SES backgrounds. The mechanisms putting youth from these—traditionally privileged—segments of society at risk for digital victimization should be explored further. After control, the differences between the IBSA group and the two other groups of victims were minor for variables such as immigrant background, having parents who are unemployed/on benefits, family break-up, parental monitoring, and support. The most distinctive differences were related to use of illegal drugs, peer networks with much substance use, and indicators of sexual behaviors and orientation. However, IBSA experiences were primarily connected to victims’ digital lives; excessive time spent on social media, and experiences of cyberbullying. With regard to PSV and the combination of PSV and IBSA, previously documented risk contexts seem to be more important.

The present study has several strengths: We use a large population-based sample with a high response rate; both digital and physical sexual violence was measured, which is unusual for studies on youth sexual victimization; and we assessed a wide range of variables enabling us to describe the characteristics of those who had experienced IBSA and PSV in detail.

However, there are limitations: First, a limitation is the use of only one item each to measure experiences of IBSA and PSV. A more comprehensive assessment would have enabled us to better capture details and dimensions of both types of victimization, and ensuing how they relate to for instance gender. Possibly, the prevalence rates of both phenomena would also have increased with a more fine-grained measure of victimization. Second, all measures were based on self-report by the adolescents, and the estimated associations with sexual victimization experiences may be influenced by common method bias (Podsakoff et al., 2003). Therefore, using multiple sources in future research is important to confirm the results from this study. Third, the cross-sectional nature of the study does not provide definite information as to whether the examined predictors are causal risk factors, just correlates, or even in some cases consequences of sexual victimization. Longitudinal studies are needed for this purpose. Fourth, even with a rather high response rate of 65% and a population-based sample, we acknowledge the possibility of non-response bias, which may have influenced our estimate of the prevalence of IBSA and PSV. Fifth, we would have benefitted from qualitative data, particularly for IBSA, such as data on the relationship between the perpetrator and victim, what kind of digital material was collected, where it happened, how it was distributed, and to whom and how the incident was perceived by the victim.

### Conclusion

The concept sexual geographies may be fruitful in drawing out some implications of our findings. It suggests that sexual outcomes are tied to the physical surroundings where they unfold (Johnston & Longhurst, 2010). Certain environments may enhance the risk of sexual victimization. Such contexts are for example parties with much alcohol (Mustaine & Tewksbury, 2002), college campuses (Sutton et al., 2019), and the nightlife scene (Tutenges et al., 2020) Most work to prevent sexual violence has focused on contexts such as these and PSV (Norwegian Government, 2020; United Nations, 2020). Less has been done regarding what we may perhaps conceptualize as digital sexual geographies. Our findings underline the need for such policies to be developed, as digital sexual violence is almost as widespread as physical sexual violence. They also underline that such policies must be gender-inclusive, given that boys and girls may be equally at risk for this type of sexual violence. More generally, prevention policies should be informed by knowledge on how online risk is produced. We follow scholars who have called for comprehensive perspectives that focus simultaneously on the digital environment, the nature and structure of platforms, and young people’s online agency and digital competence (Livingstone et al., 2015).

## Data Availability

The data may be used to replicate the analyses in agreement with NOVA/OsloMet.

## Declarations

### Availability of Data

Data are available upon request.
